# Recent advances in vasoactive intestinal peptide physiology and pathophysiology: focus on the gastrointestinal system

**DOI:** 10.12688/f1000research.18039.1

**Published:** 2019-09-12

**Authors:** Mari Iwasaki, Yasutada Akiba, Jonathan D Kaunitz

**Affiliations:** 1 Greater Los Angeles Veterans Affairs Healthcare System, Los Angeles, CA, USA; 2 Department of Medicine, David Geffen School of Medicine at UCLA, Los Angeles, CA, USA; 3 Departments of Medicine and Surgery, UCLA School of Medicine, Los Angeles, CA, USA

**Keywords:** vasoactive intestinal peptide, VIP, VPAC1, VPAC2, vasodilation, neuropeptide, gastrointestinal, gastrointestinal tract, gastrointestinal secretion, mast cells, gastrointestinal motility, colitis, ​​​​​​​functional bowel syndromes

## Abstract

Vasoactive intestinal peptide (VIP), a gut peptide hormone originally reported as a vasodilator in 1970, has multiple physiological and pathological effects on development, growth, and the control of neuronal, epithelial, and endocrine cell functions that in turn regulate ion secretion, nutrient absorption, gut motility, glycemic control, carcinogenesis, immune responses, and circadian rhythms. Genetic ablation of this peptide and its receptors in mice also provides new insights into the contribution of VIP towards physiological signaling and the pathogenesis of related diseases. Here, we discuss the impact of VIP on gastrointestinal function and diseases based on recent findings, also providing insight into its possible therapeutic application to diabetes, autoimmune diseases and cancer.

## Introduction

Vasoactive intestinal peptide (VIP) is a 28-residue amino acid peptide first characterized in 1970 that was initially isolated from porcine duodenum ^1^. A member of the secretin/glucagon hormone superfamily ^1, 2^, VIP is evolutionarily well conserved with sequence similarity among fish, frogs, and humans ^3^; among mammals, except for guinea pigs and chickens ^4^, the sequence similarity is at least 85% ^5^. VIP was initially discovered owing to its potent vasodilatory effects (as its name implies). VIP is widely distributed in the central and peripheral nervous system as well as in the digestive, respiratory, reproductive, and cardiovascular systems as a neurotransmitter and neuroendocrine releasing factor ^5, 6^. These effects contribute to an extensive range of physiological and pathological processes related to development, growth, and the control of neuronal, epithelial, and endocrine cell function. VIP has also been implicated in the regulation of carcinogenesis, immune responses, and circadian rhythms ^7^. Here, we focus on current findings related to VIP and its signals in the gastrointestinal (GI) tract with regard to its effects on secretion, intestinal barrier function, and mucosal immunology.

## Historical background

In the late 1960s, Dr. Sami I. Said at the Medical College of Virginia reported that systemic injection of extracts of mammalian lungs produced generalized vasodilation and hypotension. Together with Dr. Viktor Mutt from Karolinska University, Stockholm, Sweden, Dr. Said turned his search from the lung to duodenal extracts, which were more readily available, based on the premise that the same peptide might be present in other organs. They soon discovered that peptide fractions from porcine duodenum indeed contained a component with vasodilatory activity ^8^, supporting Bayliss and Starling’s assumption (made in 1902 during their discovery of secretin) that a “vasodepressor principle” was present in intestinal extracts ^9^.

A few years later, VIP was identified in the central and peripheral nervous systems ^10^ and has since been recognized as a widely distributed neuropeptide, acting as a neurotransmitter or neuromodulator in many organs and tissues, including the heart, lung, thyroid gland, kidney, immune system, urinary tract, and genital organs ^3^. VIP’s presence across numerous locations is related to its participation in a vast number of biological events ^11^.

## Structure and classification

**Figure 1.**
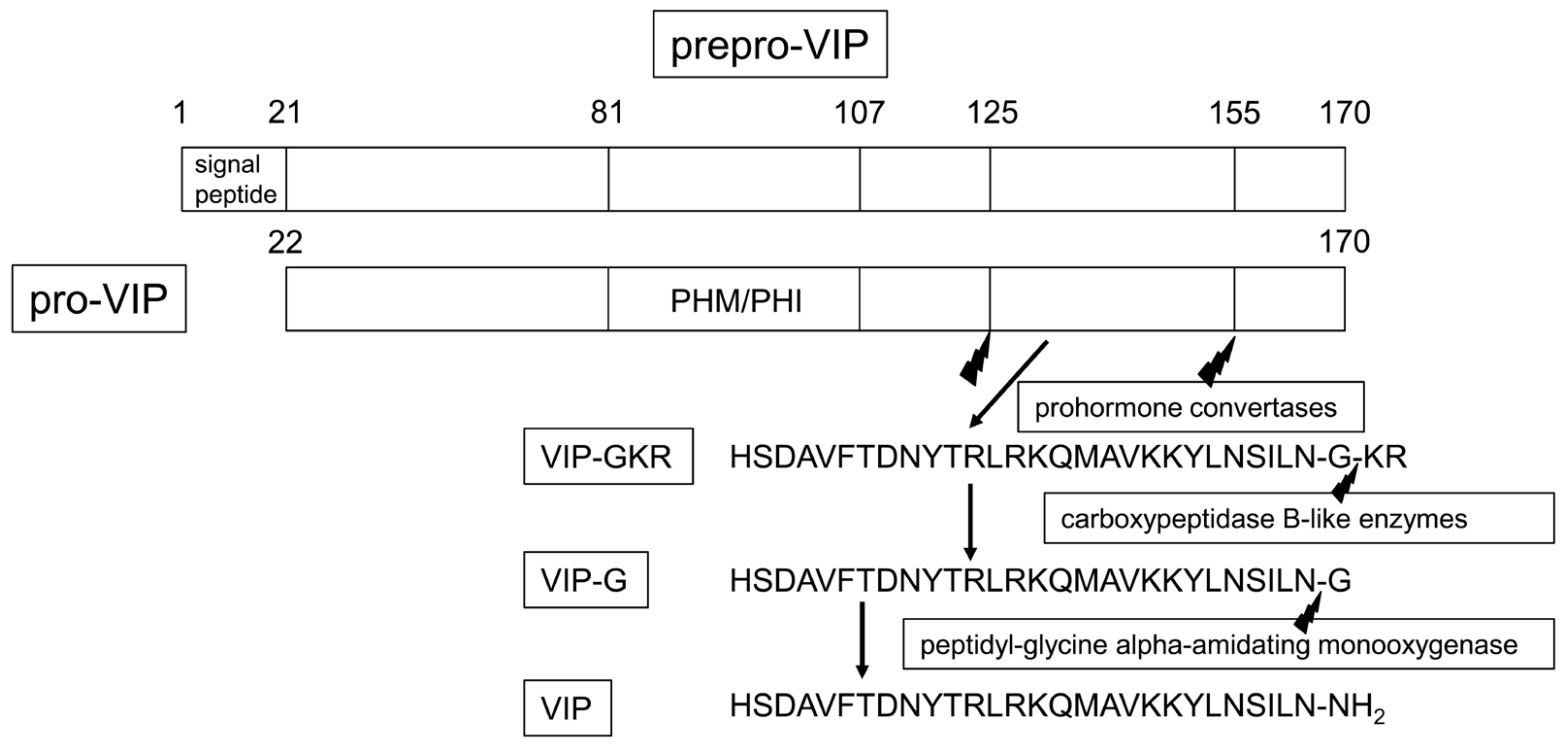
Processing of prepro-VIP to VIP. PHI, peptide histidine isoleucine; PHM, peptide histidine methionine; VIP, vasoactive intestinal peptide; VIP–GKR, VIP precursor containing the internal cleave-amidation site Gly–Lys–Arg.

The three-dimensional structure of VIP is similar to that of other members of the glucagon and secretin family ^2^, in which the structure, function, and signaling activity of pituitary adenylyl cyclase-activating peptide (PACAP) is the most closely related peptide to VIP, sharing 68% sequence homology ^11^. VIP is cleaved from a ~9 kb precursor molecule, prepro-VIP, located in the chromosomal region 6q24 containing seven exons ^6^, each encoding a functional domain. The signal peptidase located in the endoplasmic reticulum cleaves the signal peptide from the 170-amino-acid prepro-VIP, then forms a 149-amino-acid precursor peptide termed pro-VIP, which is then cleaved by prohormone convertases to a form of VIP precursor containing the internal cleave-amidation site Gly–Lys–Arg (GKR) (VIP–GKR; prepro-VIP _125–155_) ^12^ ( Figure 1). The KR residues of VIP-GKR are then cleaved by carboxypeptidase B-like enzymes to VIP-G ^13^, which is then metabolized by peptidyl-glycine alpha-amidating monooxygenase (PAM) to VIP, which has an amidated C-terminus ^11^ ( Figure 1). Prepro-VIP also contains a bioactive hormone, peptide histidine methionine (PHM) in humans or peptide histidine isoleucine (PHI) in other mammals; PHM/PHI are less potent than VIP ^14^. VIP varies its conformation depending on the environment. Most notably, its α-helical forms are present when VIP is in the presence of an anionic lipid bilayer or liposomes when bound to receptors ^5^.

## VIP and its receptors

The two receptors that recognize VIP, termed VPAC1 and VPAC2, are class B of G-protein-coupled receptors (GPCRs), also known as the secretin receptor family, which includes receptors for VIP, PACAP, secretin, glucagon, glucagon-like peptide (GLP)-1 and -2, calcitonin, gastric inhibitory peptide (GIP), corticotropin-releasing factor (CRF)-1 and -2, and parathyroid hormone (PTH). VPAC1 and VPAC2 are activated by VIP and PACAP ^15^, whereas PACAP has its own specific receptor, named PAC1, for which VIP has very low affinity ^16^. Through these receptors, VIP can mediate an extensive number of GI functions such as regulating gastric acid secretion, intestinal anion secretion, enzyme release from the pancreas, cellular motility, vasodilation, and intestinal contractility ^17– 19^. The localization of VIP, VPAC1, and VPAC2 is closely related to their physiological and pathological functions, which are also discussed under the heading “Functions in the GI tract”.

### Localization of VIP

VIP is produced in the neurons in the central and peripheral nervous systems. VIP is mainly localized in the myenteric and submucosal neurons and nerve terminals in the GI tract ^20, 21^. Endogenous VIP is released by numerous stimuli such as acetylcholine (ACh) ^22^, ATP ^23^, serotonin (5-HT) ^24^, substance P (SP) ^25^, GLP-2 ^26^, and xenin-25 ^27^ from at least two populations of VIP-positive nerves: cholinergic and non-cholinergic VIP-releasing nerves. In guinea pig small intestine, most VIP-positive nerves in the mucosa and submucosa are non-cholinergic secretomotor neurons ^28^ and well colocalized with neuronal nitric oxide synthase (nNOS) in human colonic circular muscles ^29^.

VIP is also expressed in immune cells, such as activated T cells ^30, 31^, and therefore present in lymphoid tissues including Peyer’s patches, the spleen, and lymph nodes, in addition to the VIP-ergic innervation in lymphoid tissues. VIP is produced by immune cells including T cells, B cells, mast cells, and eosinophils stimulated by lipopolysaccharide (LPS) and proinflammatory cytokines including tumor necrosis factor (TNF)-α, interleukin (IL)-6, and IL-1β ^32^.

Interestingly, VIP-positive parasympathetic nerves are present in the pancreatic islet, and vagal stimulation increases the release of VIP in the canine islet ^33^. PACAP is also present in the pancreatic islet and amplifies the glucose-induced insulin secretion ^34^. These findings suggest that VIP and PACAP modulate glucose-induced insulin secretion, similar to the incretins GLP-1 and GIP.

### Phenotype of VIP deficiency

The VIP knockout (KO) mouse exhibits phenotypes, including disturbances of circadian rhythm ^35^, inflammatory responses ^36, 37^, and metabolism ^38^. In the GI tract, VIP KO mice exhibit abnormalities of the small intestine rather than of the colon, with increased villus length, reduced mucus secretion, thickened muscle layers, and impaired GI transit ^39^. Furthermore, VIP deficiency increases small intestinal crypt depth with increased crypt cell proliferation, which is not reversible with exogenous VIP treatment ^40^. In contrast, the colon of VIP KO mice exhibits decreased crypt height with reduced cell proliferation and increased apoptosis, reduced mucus production, and increased fluorescein-dextran 4000 (FD4) permeability ^37^. Interestingly, exogenous VIP treatment of VIP KO mice restores these changes in the colon ^37^ but does not reverse the mucosal changes in the small intestine as mentioned above ^40^. Therefore, the physiological and pathological contributions of VIP towards growth and development may differ among GI segments.

### VPAC1 in the GI tract

VPAC1, for which no receptor splice variant is known, was first isolated and identified from the rat lung and later identified in human tissues. The majority of VIP actions are mediated through the VPAC1 receptor expressed on the epithelial cells, cholinergic excitatory motor neurons innervating longitudinal muscles, cholinergic secretomotor neurons, and mucosal mast cells ^41, 42^. Selective agonists and antagonists have been synthesized for its anticipated experimental and clinical use ^15, 43^.

VPAC1 in mice and humans is predominantly expressed in the colon relative to the small intestine ^44^ and is predominantly expressed in the mucosa and submucosa compared to the muscle layers in rat ileum ^27^, suggesting that VIP effects on epithelial functions, including ion transport, mucus secretion, tight junction protein expression, and cell proliferation, are mainly mediated via VPAC1 activation. VPAC1 localization in the epithelial cells is thought to be on the basolateral membranes, since serosally applied VIP increases electrogenic anion secretion in the small and large intestine ^45, 46^. Nevertheless, the exact localization of VPAC1 on the basolateral membranes of epithelial cells has not been reported, whereas VPAC1 was immunolocalized to the apical membranes of mouse and human colonic epithelial cells ^44^. Functional studies of VPAC1 activity through the apical membranes of colonocytes are awaited.

VPAC1 is constitutively expressed on T cells and macrophages but less on dendritic cells, mast cells, and neutrophils ^11^. VIP differentially induces histamine release from mast cells in that peritoneal mast cells respond to VIP more than intestinal mucosal mast cells ^47^, likely corresponding to the VPAC1 activation on mast cells.

VPAC1 KO mice exhibit impaired neonatal growth and increased post-weaning death due to intestinal obstruction and hypoglycemia, histologically with increased mucosal cell proliferation, bowel wall thickening, and smaller pancreatic islet size ^48^, suggesting that VPAC1 is essential for the normal development of the intestinal tract and the endocrine pancreas.

### VPAC2 in the GI tract

VPAC2 receptors are predominantly expressed in smooth muscles throughout the GI tract and in vascular smooth muscles in humans ^49^ and mice ^50^. Interestingly, thyroid follicles also show VPAC2-specific binding ^50^. Although VPAC2 expression in nerves and follicles in the thyroid and parathyroid is reported ^51^, there is no evidence of thyroid or PTH release by VIP or PACAP, whereas VIP increases thyroid blood flow ^52^. VPAC2 is also expressed at a high level in pancreatic β cells ^53^. VPAC2 is upregulated in activated macrophages induced by LPS and T helper (Th) cells induced by IL-4 stimulation ^11^.

VPAC2 KO mice showed significant growth impairment, decreased fat mass and increased lean mass, increased insulin sensitivity, and increased basal metabolic rate with lower serum thyroid hormone (free T3) levels and lower serum insulin-like growth factor-1 only in young females ^54^. Another group reported that VPAC2 KO mice exhibited impaired circadian rhythms with reduced metabolic rates and disrupted feeding rhythm ^55^. However, the basal phenotype of the GI tract of VPAC2 KO mice has not been reported, although VPAC2 KO mice exhibit enhanced susceptibility to chemically induced colitis ^56^. Predominant VPAC2 expression in GI smooth muscles predicts impaired intestinal motility in VPAC2 KO mice, since VIP KO mice show delayed intestinal transit ^39^. Similarly, although chemically induced colitis was less severe in PACAP KO mice ^57^, the intestinal phenotype of PACAP KO mice has not yet been reported.

## Functions in the GI tract

### Prosecretory action of VIP

VIP released from enteric nerves stimulates anion secretion from the enterocytes via G _s_-coupled VPAC1 activation ^58^, followed by adenylyl cyclase activation, increased intracellular cAMP, protein kinase A (PKA) activation, and cystic fibrosis transmembrane conductance regulator (CFTR) activation ^46, 59^. In the duodenum, exogenous VIP increases protective HCO _3_^–^ secretion via a CFTR-dependent pathway ^46^. In the ileum and colon, VIP increases electrogenic Cl ^–^ and HCO _3_^–^ secretion ^27, 45, 59^. VIP also increases Cl ^–^ secretion in porcine gallbladder ^60^ and increases porcine pancreatic fluid and HCO _3_^–^ secretion ^61^.

Hypersecretion of VIP leads to severe watery diarrhea in humans. VIP-secreting endocrine tumors termed VIPomas are the best-characterized models of increased endogenous VIP secretion. Hypersecretion of VIP by this ectopic tumor causes large-volume watery diarrhea, hypokalemia, and achlorhydria known as pancreatic cholera, the Verner–Morrison syndrome, or the WDHA syndrome ^62^, due to the action of VIP on VPAC1 receptors in the intestinal mucosa that increases Cl ^–^ and water movement into the intestinal lumen ^58^. One case report shows that a patient with WDHA syndrome was successfully treated with octreotide, a somatostatin analog, and octreotide-based radionuclide scanning localized the pancreatic tumor, which was VIP and VPAC1 positive by immunohistochemistry ^63^, suggesting that hypersecretion of VIP from a VIPoma affects tumor growth and that VIP release is modified via VPAC1 activation with positive or negative feedback. VIP and PACAP also stimulate amylase secretion from pancreatic acini of rat and guinea pig via both VPAC1 and VPAC2 activation ^64^.

### Vasodilatory action of VIP

VIP acts as a potent vasodilator. Close intra-arterial infusion of VIP increases blood flow in the gastric, small intestinal, and colonic mucosa in cats and rats ^65, 66^. In contrast, systemic intravenous (IV) infusion of VIP decreases mucosal blood flow in the rat duodenum, accompanied by systemic hypotension ^67^. Vasodilatory effects of VIP are mediated via VPAC1 activation on endothelial cells, followed by release of NO, and via VPAC2 activation on vascular smooth muscle cells in the porcine basilar arteries ^68^. Although the detailed mechanisms of VIP-induced vasodilation in the GI mucosa are not fully understood, the basilar artery study suggests that VIP-induced mucosal hyperemia may be mediated via direct activation of vascular smooth muscle VPAC2 and indirectly via VPAC1 activation with NO release. Close intra-arterial infusion of ATP increases gastric and small intestinal mucosal blood flow concomitant with parallel release of VIP ^69^, suggesting that neural ATP release and P2 receptor activation on VIP-ergic nerves may induce vasodilation via VIP release. VIP also inhibits lymphatic vessel pumping via VPAC2 activation on lymphatic smooth muscle cells ^70^, suggesting that locally released VIP modulates lymph drainage and is implicated in inflammation-associated edema.

### Smooth muscle contraction and relaxation by VIP

VIP contracts and relaxes GI smooth muscles. Rabbit and guinea pig gastric and tænia coli smooth muscle cells express only VPAC2, not VPAC1 or PAC1 ^71^. Autoradiography using a VPAC2-selective agonist demonstrated that VPAC2 is predominantly expressed on smooth muscle cells of the vasculature of the smooth muscle layers of the GI tract ^50^. Human gastric smooth muscle cells are relaxed in response to VIP, most probably via VPAC2 activation ^72^. Selective VPAC2 agonists, not VPAC1 agonists, relax pre-contracted longitudinal muscles of rat fundic stomach ^73^. In contrast, VPAC1 is expressed on the myenteric neurons colocalized with choline acetyltransferase (ChAT), and VIP contracts longitudinal muscles of guinea pig jejunum via muscarinic receptor and VPAC1 activation ^41^, suggesting that VPAC1 activation releases ACh from secretomotor neurons. PACAP-induced, non-adrenergic, non-cholinergic (NANC) relaxation of longitudinal muscle of the proximal colon is markedly reduced in PAC1 KO mice ^74^, suggesting that PAC1 expressed on NANC nerves mediates PACAP-induced relaxation and PACAP may also directly activate VPAC2 on smooth muscle cells, and then induce relaxation.

### Gastric inhibitory action of VIP

VIP inhibits gastric acid secretion via inhibition of gastrin release in dogs ^75, 76^. PACAP also inhibits gastric acid secretion stimulated by pentagastrin and histamine ^77^. A study using isolated histamine-containing enterochromaffin-like (ECL) cells and somatostatin (SST)-containing D cells demonstrate that PAC1 is expressed on ECL cells and PACAP, not VIP, increases histamine release from ECL cells, whereas D cells release SST in response to both VIP and PACAP ^78^. Furthermore, SST blockade with specific antibodies enhanced PACAP-induced gastric acid secretion in rats *in vivo* ^78^. VIP-positive and PACAP-positive nerves are present in the gastric mucosa ^79, 80^. Fluorescent protein-tagged reporter mice for SST demonstrate that purified D cells express VPAC1 and release SST in response to VIP ^81^. These results suggest that VIP inhibits gastric acid secretion via VPAC1 activation on D cells and SST release, whereas PACAP stimulates acid secretion via histamine release from ECL cells, parallel with SST release from D cells via VPAC1 activation.

### VIP effects on epithelial paracellular permeability

VIP modulates epithelial paracellular permeability via regulation of the expression and function of epithelial tight junction proteins. VIPergic pathways increase the expression of the tight junction protein zonula occludens-1 (ZO-1) in human polarized colonic epithelial monolayers co-cultured with human submucosa containing the submucosal plexus, associated with reduced epithelial paracellular permeability ^82^. VIP also ameliorates bacterial infection-induced intestinal barrier disruption by preventing the translocation of tight junction proteins ZO-1, occludin, and claudin-3 in a *Citrobacter rodentium*-induced colitis model ^83^.

Mucosal inflammation increases epithelial paracellular permeability primarily due to the alteration of the epithelial tight junction complex by TNF-α and interferon (IFN)-γ derived from activated macrophages and T cells ^84^. Since VIP and PACAP equally reduce TNF-α release from activated macrophages induced by LPS ^85^, and since VPAC2 reduces the activation of inflammatory cells ^86^, VIP–VPAC2 signaling may modify the epithelial paracellular permeability changes during intestinal inflammation.

## VIP and irritable bowel syndrome

Irritable bowel syndrome (IBS) is a chronic symptomatic GI disorder characterized by abdominal pain with altered bowel function, typically constipation and/or diarrhea. IBS with diarrhea (IBS-D) correlates with increased mast cell function and VIP release. Mast cell number and tissue immunoreactivity for substance P and VIP are greater in IBS-D patients, especially in women ^87^. A recent study shows that female IBS patients have higher plasma VIP and higher mast cell tryptase content and mast cell number in colonic biopsies compared to data from controls ^88^. Furthermore, colonic biopsies show greater transcellular bacterial passage and a higher percentage of mast cells that express VPAC1 than do biopsies from controls. Bacterial passage through the colonic biopsies was inhibitable with anti-VPAC antibodies or with the mast cell stabilizer ketotifen ^88^. These data suggest that mast cells and VIP are key modifiers of bacterial translocation in the colonic mucosa of IBS patients. However, the observations of colonic mucosal barrier function and the roles of VIP and mast cells in colonic biopsies require confirmation in patients with IBS *in vivo*.

Stress is a key factor in IBS pathogenesis. One of the stress-induced hormones is corticotropin-releasing factor (CRF), which is an important bioactive molecule not only in the central nervous system but also in the peripheral enteric nervous system. Stress-induced defecation and diarrhea in rodents is induced by peripheral administration of CRF via CRF1 receptor activation ^89^. Peripheral CRF-induced defecation and diarrhea involves VIP signals via the activation of CRF1-positive VIPergic submucosal neurons ^90^, suggesting that stress-induced diarrhea observed in IBS-D patients can be treated with VPAC1 antagonists that reduce the volume and frequency of bowel movements ^58^.

## VIP and immunity

The GI mucosa is the largest immune system in the body, likely owing to its status as the largest area of interface with the outside world. The GI tract contains luminal microbiota and numerous immune cells in the epithelium, lamina propria mucosa, and lymphoid follicles ^91^. VIP, as an anti-inflammatory mediator, downregulates the abundance of pro-inflammatory cytokines and mediators such as TNF-α, IL-6, IL-12, nitric oxide, and chemokines ^92^. VIP, which is also produced by type 2 lymphocytes (Th2), could also be classified as a Th2 cytokine ^31, 92^. The potent anti-inflammatory effects of VIP may result from its promotion of Th cell differentiation toward a “Th2” phenotype ^11^. Moreover, VIP also increases regulatory T cell production while inhibiting macrophage pro-inflammatory actions, all contributing to its anti-inflammatory effects.

VIP maintains immunological tolerance and homeostasis in the gut primarily by regulation of T cell responses and Toll-like receptor (TLR)-mediated innate immune responses. VPAC1 is primarily expressed on T cells, whereas VPAC2 expression is induced by inflammation ^92^. The anti-inflammatory effects of VIP are principally mediated via VPAC2 activation, which suppresses Th1 and Th17 functions and induces Th2 and regulatory T cells, resulting in immunosuppression ^86^. Therefore, the immunomodulatory actions of VIP expand its abilities to treat acute and chronic inflammatory and autoimmune diseases, including sepsis ^93^, multiple sclerosis ^94^, Crohn’s disease ^95^, and type 1 diabetes ^96^.

## VIP and inflammatory bowel diseases

VIP was proposed as a biomarker for inflammatory bowel disease (IBD) such as Crohn’s disease and ulcerative colitis in a study reporting elevated VIP plasma concentrations during the active inflammatory disease phase ^97^. A recent study also reported that VIP content is higher in plasma and in ileal or colonic tissues resected from Crohn’s disease or ulcerative colitis patients, respectively, than those from healthy subjects ^98^. Furthermore, the anti-inflammatory properties of VIP on Th1 immunity, which is involved in autoimmune diseases including IBD, suggest that VIP is involved in the pathogenesis of IBD and may be a therapeutic target. Nevertheless, the connection of VIP with animal models of colitis related to IBD has yet to be fully elucidated. The contribution of VIP towards the pathogenesis of dextran sulfate sodium (DSS)-induced and 2,4,6-trinitrobenzene sulfonic acid (TNBS)-induced models of colitis in mice is controversial ^18^.

The first report regarding VIP and colitis is that exogenous VIP improves TNBS-induced colitis in BALB/c mice, most likely via VPAC1 activation with anti-inflammatory and Th1–Th2 switching effects of VIP ^99^. Notably, higher doses of VIP likely aggravate the colitis ^99^. Later, another group reported that VIP administration by constant infusion enhanced the severity of TNBS-induced colitis ^100^. Subsequently, genetically modified animal models have been used to clarify the contributions of endogenous VIP and its receptors to the pathogenesis of colitis. In the DSS-induced colitis model, VPAC1 KO mice are resistant to DSS-induced colitis, whereas colitis is exacerbated in VPAC2 KO mice; PKA inhibitors reverse the impairment of DSS colitis in VPAC2 KO mice, suggesting that enhanced VPAC1 activity in VPAC2 KO mice may aggravate DSS colitis ^56^ or is alternatively explained by the protective effects of VPAC2 during the development of DSS-induced colitis, since VPAC2 activation inhibits Th1 signals ^11^.

In VIP KO mice, DSS treatment had no effect on colitis in males, compared to wild-type males, whereas body weight loss and disease activity index in females was less frequently observed in VIP KO subjects ^40^, suggesting that VIP may have enhanced pro-inflammatory functions in females. Furthermore, male VIP KO mice or wild-type mice treated with a pan-VIP receptor antagonist (VIP-hybrid ^101^) or the selective VPAC1 antagonist (PG97-269) ^15^ are resistant to DSS-induced colitis with reduced levels of colonic inflammatory mediators and cytokines ^102^, suggesting that VIP acts as a pro-inflammatory mediator. In TNBS-colitis, VIP KO mice are resistant to colitis with lower levels of TNF-α and IL-6 ^103^. Similar resistant phenotypes are observed in a VIP KO with LPS-induced endotoxemia model, where LPS induced less mortality in VIP KO mice ^36^, and with the experimental autoimmune encephalomyelitis (EAE) model, where clinical scores were less in VIP KO mice ^104^. Nevertheless, VIP KO mice develop more severe colitis in the DNBS- or DSS-induced colitis models, which is rescued by exogenous VIP treatment ^37^. Most recently, a recombinant stable VIP analog (rVIPa) was reported to ameliorate TNBS-induced colonic injury and inflammation, effectively preserving intestinal mucosal barrier function in rats ^105^, most likely owing to increased stability of the VIP analog.

These discrepancies between anti-inflammatory and pro-inflammatory effects of VIP on chemically induced colitis models may reflect the differences between endogenous and exogenous effects of VIP due to dose effects and peptide stability in the tissues and circulation because VIP is rapidly degraded by dipeptidyl peptidase 4 (DPP4), similar to the incretins ^106^, and by other peptidases. Furthermore, genetic deficiency of VIP or VPAC irreversibly alters epithelial, neural, and immune responses during development. Another possibility is that targets of VIP may induce opposite effects on inflammation; VPAC2 activation on T cells shifts Th1 to Th2 differentiation as anti-inflammatory, whereas VPAC1 activation of epithelial cells increases anion and water secretion, with resultant diarrhea, which may affect the disease activity of colitis. VPAC2 activation of GI smooth muscles increases GI motility, whereas impaired motility in VIP KO or VPAC2 KO may affect GI transit, affecting the exposure time to luminal toxic chemicals such as DSS in drinking water. Therefore, cell-specific, conditional knockout will clarify these contradictory results.

## VIP/PACAP and diabetes

The metabolic syndrome, including type 2 diabetes and obesity, is also a GI-related disorder, since insulinotropic hormones, termed incretins, including GLP-1 and GIP, are secreted from enteroendocrine L and K cells, respectively. As mentioned above, vagal stimulation increases the release of PACAP and VIP in pancreatic islets, suggesting that PACAP and VIP modulate insulin secretion from β cells through the activation of cognate receptors.

Pancreatic islet β cells express PAC1 and VPAC2 with less VPAC1 ^28^. Selective VPAC2 agonists are insulinotropic, similar to PACAP and GLP-1, amplifying glucose-induced insulin secretion ^107^. VIP KO mice exhibit elevated plasma glucose, insulin, and leptin levels with no change in islet mass ^38^, probably due to the compensatory effect of PACAP. In VPAC2 KO mice, glucose-induced insulin secretion is decreased with no change in glucose tolerance. VPAC1 KO mice show growth retardation, intestinal obstruction, and hypoglycemia ^48^, suggesting that VPAC1 is also involved in glucagon secretion, which counteracts the hypoglycemic effects of insulin. In isolated perfused pancreas, PAC1 KO mice exhibit a 50% reduction of the PACAP-induced insulin secretory response, whereas VIP-induced insulin secretion is unchanged ^108^, suggesting that the insulinotropic action of PACAP is partially mediated by PAC1. Therefore, VPAC2 agonists and PAC1 agonists are candidates for the therapy of type 2 diabetes.

## VIP/PACAP and cancers

Human cancers including bladder, breast, colon, liver, lung, pancreatic, prostate, thyroid, and uterine cancers often overexpress VPAC1, whereas VPAC2 is limited in stromal tumors such as gastric leiomyomas, sarcomas, and neuroendocrine tumors ^109^. Since VPAC1 is normally expressed in the epithelium and VPAC2 in smooth muscle in the GI tract, these expression profiles may reflect their tumor expression with VPAC1 in adenocarcinoma and VPAC2 in stromal tumors. PAC1 is also expressed in diverse tumors including brain, breast, colon, lung, neuroendocrine, pancreas, pituitary, and prostate tumors as well as neuroblastomas ^110^. This suggests that VIP/PACAP may affect tumor growth and differentiation. VIP and PACAP stimulate the growth of several cancer cell lines *in vitro* ^110^, supporting this hypothesis.

Regarding the GI tract, colon cancer tissue overexpresses VPAC1: in 35% of well-differentiated, 65% of moderately differentiated, and 87% of poorly differentiated colon cancers ^111^, predicting tumor differentiation can be accomplished by measuring VPAC1 levels. Therefore, VPAC1 can be a target for anti-cancer drugs, since VPAC1 antagonists inhibit the growth of colonic cancer cell lines *in vitro* ^112^.

Overexpression of VPAC and PAC1 in tumors can be used for imaging and targeting tumors using radiolabeled VIP analogues. Clinical studies show that radiolabeled VIP analogues localize breast cancer, pancreatic cancer, intestinal adenocarcinomas, neuroendocrine tumors, and colorectal cancer using a combination of positron emission tomography (PET) and computed tomography (CT) scans ^110, 113^. Furthermore, VIP-conjugated nanoparticles have been developed to deliver the cytotoxic drug to tumor cells overexpressing VPAC ^114^.

## Therapeutic potential of VIP

Since VIP contributes to important physiological functions including anion secretion, the regulation of permeability of epithelial tight junctions, mucosal inflammation, glycemic control, Th1–Th2 balance, and tumor growth, VIP has been suggested to be a therapeutic target for diseases such as diarrhea ^58^, IBD ^95^, diabetes ^28^, autoimmune diseases ^115^, neurodegenerative disorders ^116^, lung disease ^117, 118^, sarcoidosis ^119^, and cancers ^114^. Although VIP has well-studied anti-inflammatory and other therapeutic potential, VIP-based drug design has not been entirely successful because rapid degradation of the peptide limits its bioavailability and delivery. Furthermore, multiple cellular targets that bind VIP at high affinity may cause undesirable adverse effects. Therefore, synthesis of a stable VIP analog, or the targeted delivery of VIP or its analogs via nanoparticles are desirable options.

Recent advances in the field include the synthesis of stable analogs such as lipophilic or peptide VIP derivatives that mimic the activity of native VIP ^120^. Another strategy is VIP self-associated with sterically stabilized micelles, which protects VIP from degradation and inactivation ^115^. Injection of VIP-induced regulatory dendritic cells ameliorates TNBS-induced colitis models in mice ^95^. VIP gene transfer using lentivirus is also useful to induce immunosuppression in the murine arthritis model ^121^. Finally, VIP-tagged nanoparticles may be a useful strategy for selective drug delivery to VPAC-overexpressing tumor cells and immune cells ^114, 122^.

## Summary and conclusions

**Figure 2.**
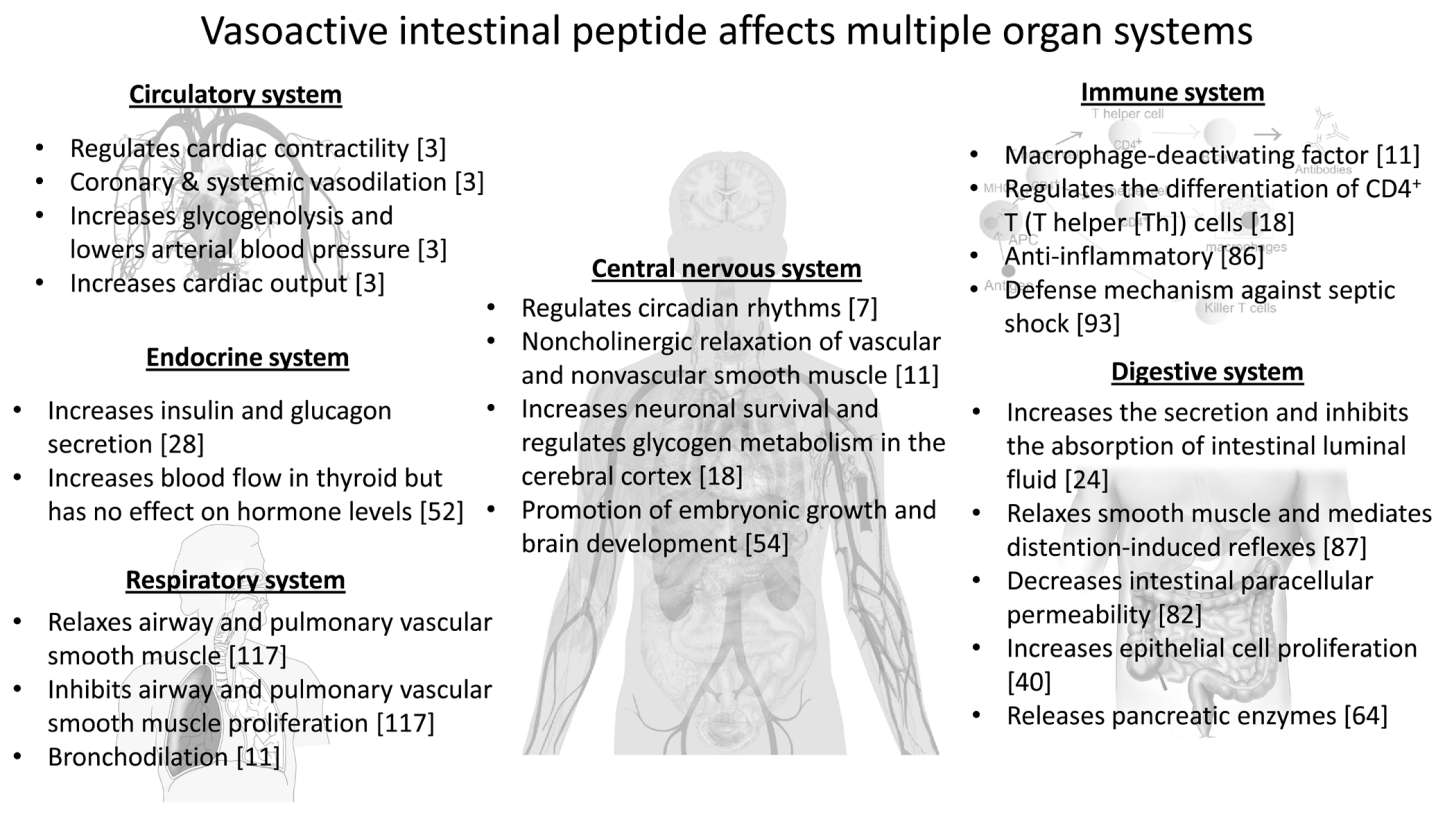
Broad multiple functions of vasoactive intestinal peptide in various organs. Number in parenthesis represents the corresponding reference number.

Since its discovery in 1970, VIP has been studied in numerous organ systems including the gastrointestinal, respiratory, cardiovascular, immune, endocrine, and central and peripheral nervous systems, where it exerts numerous important effects ( Figure 2). Nevertheless, owing to its protean and widespread effects on numerous organ systems combined with its inherent instability, VIP has been challenging to clearly discern and analyze regarding its influence on isolated pathophysiological functions. Specifically, in the gut, VIP has therapeutic potential for a variety of inflammatory disorders such as IBD. The recent progress of VIP-related medicine is aimed at improvement of its stability, selectivity, and efficacy with reduced adverse effects. In order to make optimal therapeutic use of, it is essential to further study its localization and actions, working towards selective targeting or individual effects.
